# Configuration analysis of negative silence in college classroom based on FSQCA method

**DOI:** 10.1038/s41598-025-09608-5

**Published:** 2025-07-09

**Authors:** Jian Chen, Baozhong Ye, Haifeng Miao

**Affiliations:** 1 Guilin University of Aerospace Technology, Guilin, China; 2 The Tenth Affiliated Hospital of Southern Medical University (Dongguan People’s Hospital), Dongguan, China

**Keywords:** College students, Negative silence in classroom, Antecedent conditions, Configuration perspective, FSQCA method, Human behaviour, Psychology

## Abstract

Currently, negative silence among students in college classroom is quite common. However, existing research on this phenomenon tends to analyze the net effects of antecedent conditions on outcome. In fact, negative silence in college classroom is the result of multiple factors working together. Therefore, this paper takes 222 college students as research samples and uses FSQCA method to analyze the relationships between five antecedent conditions (self-efficacy, fear of negative evaluation, interest, verbal immediacy, and nonverbal immediacy) and negative classroom silence from the configuration perspective. The results indicate that none of these conditions is necessary for the negative silence. On the contrary, three configurations of conditions could lead to this issue. The first configuration contains self-efficacy, fear of negative evaluation, and verbal immediacy. The second configuration includes self-efficacy, fear of negative evaluation, and nonverbal immediacy. The third configuration consists of self-efficacy, interest, verbal immediacy, and nonverbal immediacy. The research results of this article reveal the related factors and their configurations of negative silence in college classroom, which can help to gain a comprehensive understanding of the reasons for the phenomenon and provide references for breaking it. For instance, in teaching and learning process, it is necessary to use methods such as encouragement and appreciation to promote the self-efficacy of college students, provide effective guidance for college students to overcome the fear of negative evaluation, combine more real-life examples with knowledge to enhance college students’ learning interest, and use more verbal and nonverbal immediacy behaviors to attract the attention of college students in the classroom, so as to achieve an active and effective learning atmosphere.

**Supplementary Information:**

The online version contains supplementary material available at 10.1038/s41598-025-09608-5.

## Introduction

The cultivation of high quality college students is an essential issue for higher education to achieve sustainable development^1^. As the fundamental foothold of higher education, classroom is a vital factor affecting the quality of talent cultivation and education in colleges. In classroom, students mainly have two different learning states, namely active participation and silence. Active participation refers to ‌energetically participate in class, such as asking question to the teacher or answering the question raised by the teacher^2^. Maintaining silence manifests as silently contemplating the teacher’s raised question or showing indifference in the course learned in class^3^. When students quietly think deeply the question proposed by the teacher, it would be fine. However, in college classroom, the phenomenon of students being silent, where the teacher gives lectures seriously while students remain indifferent, or divert their attention, or lower their heads without saying a word could be seen frequently. The entire classroom presents an aphasia environment, which is a typical state of negative silence.

Negative silence in classroom is not a sound phenomenon^4^, and it has adverse effects on both teacher and students. On one hand, negative silence can cause teacher to feel low during lectures, reduce the teacher’s teaching enthusiasm, and affect the teacher’s teaching performance in the classroom. On the other hand, a dull classroom atmosphere can hinder the cultivation of students’ thinking abilities and the formation of healthy psychology. Therefore, as a non-benign form of classroom feedback, negative silence directly affects the overall quality of teaching and learning. If college students generally exhibit negative silence in the classroom, then the quality of their learning is worrying. So, in order to improve the effectiveness of classroom teaching and the quality of education, an essential issue that must be faced is how to break the negative silence phenomenon^5^. As a result, it is necessary to conduct research on negative silence, strive to understand the reasons, and take appropriate measures to address this issue.

Existing literature has conducted extensive studies on negative silence in college classroom, and many scholars have proposed the related factors of this silence. For example, previous study has shown that negative silence in the classroom is related to students’ self-efficacy, and the lower the self-efficacy, the higher the silence^6^. Besides, literature has also indicated that students’ fear of negative evaluation could significantly predict the negative silence^7^. In addition, lack of interest among students in the classroom is an essential cause of silence^8,9^. According to the research of Karim and Shah^10^, teacher’s verbal immediate behaviors have a significant relationship with students’ silent behavior during classroom hours. Tang and Wang suggested that teacher’s nonverbal immediate behaviors such as smiles and hints could make students feel cared for and warm^11^, thereby helping to break the negative silence in the classroom. Previous research has conducted net effect analysis through regression or other methods, which can obtain the relationship between a single condition and outcome. From the perspective of general systems theory^12^, the reasons or conditions for generating social phenomena are interrelated rather than independent. Therefore, method of configuration should be adopted to explain social phenomena^13^. Negative silence in college classroom is a complex phenomenon caused by multiple factors, and researchers should analyze it from the perspective of configuration. However, existing literature has not explored the combined effects of conditions on this phenomenon from configuration perspective. Consequently, this study attempts to break through the limitation of net effects analysis and uses the configuration perspective to study and identify the relationships between five factors (self-efficacy, fear of negative evaluation, interest, verbal immediacy, and nonverbal immediacy) and negative silence in college classroom. Specifically, this article takes college students as the research objects, and then FSQCA (Fuzzy-Set Qualitative Comparative Analysis) method is used to analyze the configuration effects of those five factors on the phenomenon after data collection. At the same time, corresponding countermeasures are put forward to prevent or at least reduce the negative silence. This article aims to explore how the combinations of multiple conditions lead to negative classroom silence, and FSQCA is a professional method for studying the combined effects of multiple factors. Therefore, it is appropriate for this paper to choose the FSQCA method for research. According to the configuration results obtained by FSQCA method, the conditions included in the configurations that lead to negative silence in classroom could be acquired, which is helpful to effectively understand the reasons for the negative silence and take corresponding interventions to eliminate it.

The arrangements of the later parts of this paper are as follows: Firstly, literature review was conducted. Secondly, the method and data were introduced. Then, FSQCA method was used for empirical analysis. Finally, the conclusion section was presented.

## Literature review

### Attribution theory

Attribution theory states that human behaviors are caused by internal and external factors^14,15^. The internal factors mean the factors of the actor self, such as self-efficacy, while the external factors refer to the factors outside, like the influence of others. Attribution theory pioneers the attribution issue and provides the basis of how to explain human behaviors based on causal inference, which has far-reaching influence^16^. In the past time, attribution theory has been widely used in multiple fields, including management^17^, medicine^18^, and education^19^. Based on Attribution theory, this article tries to explain the negative silence among college students in the classroom according to the internal and external factors. Specifically, the internal factors are students’ self-efficacy, fear of negative evaluation and interest, and the external factors are the teacher’s verbal immediacy and nonverbal immediacy.

### Self-efficacy

The concept of self-efficacy was first proposed by Bandura^20^, which refers to the individual judgment on whether he or she has the ability to complete a certain behavior. Self-efficacy is seen as a positive psychological factor rather than a negative one, and it may have a buffering effect on negative psychological factors^21^. In college classroom, students with strong self-efficacy believe that they can understand and master the knowledge taught by the teacher^22^. Besides, they have the confidence to answer the question raised by the teacher well. Consequently, when teacher requires students to engage in discussion or other activities, they will actively participate. The higher students’ sense of self-efficacy, the more active they will be in learning, thus reducing their tendency to silence in class^23^. Conversely, lower self-efficacy prompts students to engage in negative silence^24^. Students that are lack of self-efficacy may tend to remain silent and will not show any positive classroom participation because they do not have enough confidence to face such things in the classroom. Therefore, this paper suggests that self-efficacy is a factor related to negative silence in college classroom. Then, self-efficacy is selected as a condition.

### Fear of negative evaluation

Social anxiety is, to some extent, a response of people to negative evaluations of others^25^. Fear of negative evaluation refers to the individual fear of bad evaluations from others outside and the pain they feel towards these evaluations^26^. So, to avoid this type of fear, people may choose to keep silent. College students are sensitive in their hearts, very concerned about external evaluations, and also afraid of making a fool in front of others and becoming objects of ridicule^27^, which can undermine their self-confidence and self-esteem^28^. In college classroom, if students actively answer the teacher’s question or ask the teacher, they may worry that such behaviors will be negatively evaluated by others that will make them feel uncomfortable. For example, other students may perceive such behaviors as the way to showcase themselves or intentionally disrupt the classroom order. As a result, instead of the possibility of receiving such negative evaluations, students choose to remain silent. From the perspective of this, fear of negative evaluation is related to negative silence in college classroom. Consequently, this study ‌incorporates‌ fear of negative evaluation as a condition.

### Interest

Interest in educational psychology is often described as a dynamic relationship between a person and an object, which can be a subject, idea, activity, and event^29^. When individuals are interested in something, they feel pleasure in doing it^30^. Interest has always been a significant subject in educational research. More than 100 years ago, the educator Dewey recognized the importance of interest^31^, and since then, academic community has begun to study it. Students’ interest in classroom reflects their positive emotions and cognitions in the learning process and is considered to be a vital factor in the learning environment^32^. Research has shown that interest is positively correlated with learning, and active interest helps students focus on and complete learning tasks^33^. On the contrary, individuals who have no interest in learning will not actively participate in class, and their thoughts will not flow along with the teacher’s ideas. As a consequence, their negative silence occurs. In the classroom led by the teacher, college students are not given sufficient time and opportunities to express themselves, resulting in the loss of interest and classroom silence^34,35^. Moreover, studies have shown that the loss of students’ interest in learning and the difficulty in stimulating their interest are the vital causes of classroom silence^9,36^. Then, interest is selected as a condition.

### Immediacy

Immediacy stands for the perceived psychological or physical closeness between people, and teacher’s immediate behaviors in class can shorten the perceived distance between the teacher and students^37^. Like other interpersonal relationships, the interaction between teaching and learning is realized through explicit and implicit communication. Interpersonal perception and communication between teacher and students are very important for the teaching process, and immediacy is a critical variable in this relationship^38,39^. In educational environment, the immediacy of teacher is closely related to learners’ emotions and behaviors and can enhance learners’ inspiration^40^. Previous study has shown that teacher’s immediate behaviors could motivate students to participate in the classroom^41^, which can effectively break the negative silence^42^. If the teacher could make good use of immediacy, learners’ enthusiasm for learning can be stimulated and maintained. Generally, teacher’s immediacy includes verbal immediacy and nonverbal immediacy, both of which may contribute to cultivating a close and friendly relationship between the teacher and students^43^. Also, the two kinds of immediacy could lead to students’ increased affinity and learning effectiveness^44^.

Verbal immediacy can be directly used by teacher and plays an important role in students’ satisfaction or dissatisfaction with the teacher and course. In practice, teacher’s verbal immediacy generally includes humor, calling names, providing personal examples, sharing experiences^45^. Students are generally interested in teacher’s verbal immediacy, and the appearance of this immediacy can attract students’ attention in class to a certain extent, thus achieving better learning effect and teaching quality. Existing literature suggests that teacher’s verbal immediacy such as positive classroom evaluation is crucial for students’ learning behaviors^46^. Also, positive and immediate language evaluation of teacher could enhance students’ confidence and initiative in the classroom^47^, and increase students’ enthusiasm for raising their hands to speak^48^. Even if students are unsure whether they are correct, they will be willing to try answering question in class because of the teacher’s encouragement and trust^49^. Besides, teacher’s verbal immediacy implies some oral information, which shows sympathy, friendliness, praise, etc. and can to some degree make students feel relaxed and more positive, thereby stimulating students to participate in communication and learning^50^. However, when there is no such verbal immediacy, students’ attention will not be well attracted, and they will not actively participate in the class, thus showing negative silence. Then, verbal immediacy is selected as a condition.

Nonverbal immediacy generally uses indirect behavioral signs to produce immediacy. For instance, it contains body language, gesture, facial expression, and body movement^40^. All these nonverbal immediacy can achieve certain effects through nonverbal means. For example, teacher’s facial serious expression is helpful to create good classroom order and atmosphere^11^. Nonverbal behaviors of teacher in classroom can determine whether the teacher and teaching content would be well received by students^44^, which is associated with a variety of positive outcomes^37^. There are many characteristics of nonverbal immediacy in practical performance, such as constructive nodding, physical distance, grin, eye contact^51^. The presence of these types of nonverbal immediacy can, of course, make the students more energetic and attentive to the class and promote better participation of them, thus breaking the negative silence. On the contrary, when teacher does not use these types of nonverbal immediacy in classroom and students do not feel them, the classroom is more likely to become passive and silent. Then, nonverbal immediacy is selected as a condition.

**Fig. 1:**
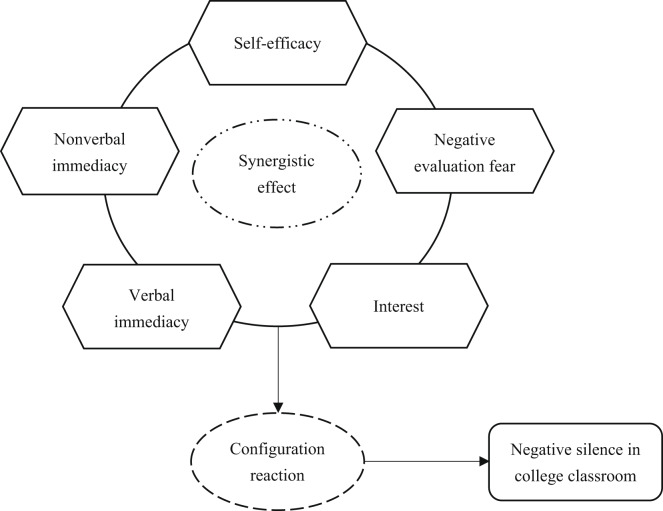
Conceptual model.

Based on the analysis above, relationships between different conditions and negative classroom silence have been explored from the perspective of a single variable. According to general systems theory^12^, the conditions for causing social phenomena are interrelated. Ragin also suggests analyzing social phenomena from configuration perspective^13^. Classroom negative silence has passive impacts on students’ learning outcomes and even the educational industry, but it is currently unclear what configuration of conditions leads to this issue. Therefore, it is necessary to analyze the silence from the perspective of configuration and explore how multiple conditions combine to bring about this phenomenon. On one hand, compared with existing literature based on a single condition, it can theoretically enrich the research of the reasons for the negative silence in classroom. On the other hand, in practice, revealing the reasons for students’ negative silence from configuration perspective is more in line with the actual situation, and then taking relevant intervention measures based on configuration reasons to eliminate this silence could produce better effects. Consequently, a conceptual model can be constructed according to configuration perspective, as shown in Fig. 1 below.

From Fig. 1, it is known that the combined effects of multiple conditions generate negative silence in college classroom. The comprehensive effects may from the combination of two, three, four, or even five conditions, which needs further verification. Taking the combination of three conditions as an example, which are self-efficacy, fear of negative evaluation, and interest. This combination indicates that when these three conditions work together, negative classroom silence will occur. In other words, negative silence occurs among college students who have low self-efficacy, are afraid of negative evaluation and lack of interest. Based on the research purpose of this article and the core idea of FSQCA method, two hypotheses are proposed as follows.

#### Hypothesis 1

Negative silence in college classroom is generated by the combined effects of multiple conditions among self-efficacy, fear of negative evaluation, interest, verbal immediacy, and nonverbal immediacy.

#### Hypothesis 2

There may be more than one combination among the five conditions that could lead to the silence.

## Method and data

### QCA method

Qualitative Comparative Analysis (QCA) considers the case as a configuration of conditions and analyzes whether conditions and their combinations are sufficient for result^52^. QCA aims to identify the causal relationships between conditional configurations and result and answer the question of what conditional configurations can lead to the occurrence of the result^53^.

Firstly, the idea that a single condition can produce result is not supported by QCA, which proposes the assumption of concurrent causal relationships, that is, multiple conditions can only produce result when they occur simultaneously. Secondly, a given combination of conditions may not be the only way to produce a certain result, and other combinations of conditions may also produce the same outcome. Again, causal effects no longer have consistency. On the contrary, a given condition may have an impact on result when combined with certain conditions while not necessarily when combined with other conditions. Finally, QCA no longer assumes symmetry in causal relationships, but rather assumes asymmetry^54^.

According to the types of variables, QCA can be divided into Crisp-Set Qualitative Comparative Analysis (CSQCA), Multi-Value Qualitative Comparative Analysis (MVQCA), and Fuzzy-Set Qualitative Comparative Analysis (FSQCA). FSQCA can solve the issue of partial membership, allowing researcher to calibrate variables to any value between 0 (completely non-membership) and 1 (completely membership) during data calibration. As a result, FSQCA has been used in a wide range of fields in recent years, such as education^53^, psychology^55^, and business^56^. Because the aim of this article is to explore how five conditions (self-efficacy, fear of negative evaluation, interest, verbal immediacy, and nonverbal immediacy) collectively lead to negative silence in college classroom, and the variables in this article do not belong to 0 or 1, FSQCA is used for analysis.

### Data collection

This study adopts the FSQCA method to reveal the configuration causes of negative silence in the classroom of college students. Based on previous research in the literature review section, this paper constructs a theoretical model to clarify the relationships between conditions and result, as shown in Fig. 1. FSQCA is a case-based research method, and the key of using it for research is to select appropriate empirical data. The authors conducted data collection through questionnaire survey, in which all items of each variable were based on references to existing literature. The research was in line with the Declaration of Helsinki, and the approval was obtained from the ethics committee of the Tenth Affiliated Hospital of Southern Medical University (Dongguan People’s Hospital) (Approval Number: G-2023032). Participants were informed of the study’s objective, assured of the participation principles of voluntariness and anonymity, and advised of their rights to withdraw at any time. Informed consents were obtained from all participants prior to the research.

The items of self-efficacy were adapted from previous study of Steigen et al.^57^, and four items were included, which were (1) I am not confident that I could deal efficiently with unexpected events. (2) When facing difficulties, I do not think I have good coping capabilities. (3) If someone opposes me, I can’t find the means and ways to get what I want. (4) If I am in trouble, I can’t usually think of a solution. Participants were asked to judge on a 5-point scale: 1 = strongly disagree, 2 = disagree, 3 = neutral, 4 = agree, and 5 = strongly agree. For the items, higher scores indicated higher lack of self-efficacy. In the current study, Cronbach’s α coefficient for self-efficacy was 0.81, indicating favorable reliability.

Fear of negative evaluation referred to the research of Weeks et al.^58^, and it included five items: In class, if I answer the teacher’s question or communicate with classmates, (1) I am afraid that they will not approve of me. (2) I am afraid that they will find fault with me. (3) I am usually worried that I will leave a bad impression on them. (4) I am often worried that I may say the wrong thing. (5) I am worried about what they may be thinking about me. Participants rated the items on a 5-point scale, which are 1 = strongly disagree, 2 = disagree, 3 = neutral, 4 = agree, and 5 = strongly agree. For the items, higher scores indicated higher fear of negative evaluation. In this study, Cronbach’s α coefficient for fear of negative evaluation was 0.93, representing favorable reliability.

Interest was assessed adapted from previous study of Kleespies et al.^59^, and it contained three items: (1) I think the content taught by the teacher in class is not fun. (2) I would not want to learn more about the content taught by the teacher in class. (3) The content taught by the teacher in class is not important to me. Each item was answered by participants on a 5-point Likert scale (1 = strongly disagree, 2 = disagree, 3 = neutral, 4 = agree, and 5 = strongly agree), and higher scores indicated higher lack of interest. In the current study, Cronbach’s α coefficient for Interest was 0.77, indicating favorable reliability.

Verbal immediacy was assessed adapted from previous research of Gorham^60^. The three items of this variable were as follows: (1) The teacher does not use personal examples or talk about experiences he/she has had outside of class. (2) The teacher does not select students by name to answer question in class. (3) There is no collective discussion in class. Items were answered using a 5-point Likert scale (1 = strongly disagree, 2 = disagree, 3 = neutral, 4 = agree, and 5 = strongly agree). Higher scores indicated higher lack of verbal immediacy. In the current study, Cronbach’s α coefficient for verbal immediacy was 0.65, indicating acceptable reliability based on the research of Ogilvie et al.^61^, Setbon and Raude^62^, and Rauzana and Dharma^63^.

Nonverbal immediacy was assessed adapted from the study of Gorham^60^. The three items were (1) The teacher does not use any gestures while talking to class. (2) The teacher’s vocal expression is monotonous during the class. (3) The teacher does not move around the classroom while teaching. Participants were asked to judge on a 5-point scale: 1 = strongly disagree, 2 = disagree, 3 = neutral, 4 = agree, and 5 = strongly agree. Higher scores of items implied higher lack of nonverbal immediacy. In the current study, Cronbach’s α coefficient for nonverbal immediacy was 0.67, meaning acceptable reliability according to the research of Ogilvie et al.^61^, Setbon and Raude^62^, and Rauzana and Dharma^63^.

Negative silence in classroom was assessed adapted from previous study of Liu^64^, and it had four items: In class, (1) I will not actively respond to the teacher. (2) I will not ask the teacher for question. (3) I will not question the views put forward by the teacher. (4) I try not to make eye contact with the teacher. Participants rated the items on a 5-point scale, which are 1 = strongly disagree, 2 = disagree, 3 = neutral, 4 = agree and 5 = strongly agree. Higher scores of items implied higher negative silence. In this study, Cronbach’s α coefficient for negative silence in classroom was 0.79, indicating favorable reliability.

It should be noted that all the questionnaire items were scored positively. The higher scores of the conditional variables (self-efficacy, fear of negative evaluation, interest, verbal immediacy, and nonverbal immediacy), the more likely they were to cause negative silence. The higher score of the outcome variable (negative silence in classroom), the more severe the negative silence.

This article used the simple random sampling method to collect data according to the natural classroom with the silence phenomenon, and the sample size was 222. Participants were students from six universities (Guilin University of Aerospace Technology, Guangxi University of Science and Technology, Guilin Tourism University, Guangxi University of Finance and Economics, Guilin University of Electronic Technology, and Guangxi Minzu University) in Guangxi Province, China, and among them, the number of male college students was 65, while the number of female students was 157, accounting for 29.28% and 70.72% of the total sample. There were 120 freshmen, 34 sophomores, 33 juniors, and 35 seniors, accounting for 54.05%, 15.32%, 14.86%, and 15.77%, respectively. All of the participants were over 18 years old, and they participated in this survey voluntarily and anonymously. Their participation indicated that they were aware of this survey and agreed to all matters related to this study. It should be pointed out that according to existing literature, male students are more active in speaking in the classroom compared to the female^65^. Because female students are introverted and reserved in the classroom^66^, they tend to be more inclined to remain silent^67,68^. In the sample of this article, the larger proportion of female students is consistent with that view. Indeed, the gender imbalance in the sample size of this study may limit the generalizability of the results, thus, future research should consider collecting gender balanced sample for exploration.

The descriptive statistics of variables is shown in Table 1. It is worth mentioning that the measurement results of each variable are the average of its all items in the questionnaire.

**Table 1:** Descriptive statistics results.

Descriptive indexes	Conditional variables					Outcome variable
	Self-efficacy	Fear of negative evaluation	Interest	Verbal immediacy	Nonverbal immediacy	Negative silence in classroom
Average value	2.68	2.79	2.26	2.59	2.23	2.94
Standard deviation	0.74	0.90	0.70	0.72	0.68	0.72
Maximum value	5.00	5.00	4.33	5.00	4.33	5.00
Minimum value	1.00	1.00	1.00	1.00	1.00	1.00

### Tests of reliability, validity, and common method biases

Before data analysis, it is necessary to test the reliability and validity of the data. For the current data, the overall Cronbach’s α coefficient is 0.88, with Cronbach’s α coefficients exceeding 0.65 (self-efficacy: 0.81; fear of negative evaluation: 0.93; interest: 0.77; verbal immediacy: 0.65; nonverbal immediacy: 0.67; negative silence in classroom: 0.79) and Composite Reliabilities (CR) exceeding 0.75 (self-efficacy: 0.82; fear of negative evaluation: 0.92; interest: 0.83; verbal immediacy: 0.75; nonverbal immediacy: 0.77; negative silence in classroom: 0.82) for each variable. Through factor analysis, the Kaiser-Meyer-Olkin (KMO) value is 0.85, and the cumulative variance contribution rate is 68.47%. Furthermore, all items displayed factor loadings exceeding 0.54 (items of self-efficacy: 0.71, 0.80, 0.62, and 0.76; items of fear of negative evaluation: 0.80, 0.80, 0.87, 0.84, and 0.83; items of interest: 0.72, 0.85, and 0.80; items of verbal immediacy: 0.63, 0.89, and 0. 60; items of nonverbal immediacy: 0.70, 0.75, and 0.73; items of negative silence in classroom: 0.80, 0.86, 0.69, and 0.54), and the minimum Average Variance Extracted (AVE) of the variables is 0.51 (self-efficacy: 0.53; fear of negative evaluation: 0.69; interest: 0.63; verbal immediacy: 0.51; nonverbal immediacy: 0.53; negative silence in classroom: 0.54). Therefore, the results of reliability and validity tests are acceptable.

Due to the potential for common method bias in collecting data through self-report, conducting a test is needed. At first, Harman’s single factor test is carried out, and the result shows that the maximum factor variance explained rate is 30.21%, which is less than the critical value of 40%^69^. Then, this article compares the multi-factor model and single-factor model for this test. Specifically, the fit of six-factor model (CMIN = 418.01, CMIN/DF = 2.16, SRMR = 0.06, IFI = 0.90, CFI = 0.90, RMSEA = 0.07) is significantly better than the single-factor model (CMIN = 1123.48, CMIN/DF = 5.38, SRMR = 0.11, IFI = 0.58, CFI = 0.58, RMSEA = 0.14). As a result, the common method variance of measures in this article is minimal.

### Data calibration

Using FSQCA method for research requires calibration of data, namely, converting crisp data into more accurate but less precise set values^70^, so that data is transformed into a set concept. Referring to existing literature, this paper sets the three calibration points of complete membership, intersection, and complete non-membership to 0.90, 0.50, and 0.10, respectively, which are referenced the research of Greckhamer^71^. In addition, to avoid the configuration attribution problem where the membership degree of the antecedent case is exactly 0.50, this paper subtracts the 0.001 constant from the 0.5 membership degree^72^. The calibration anchor points for each variable are shown in Table 2.

**Table 2:** Calibration anchors for variables.

Calibration anchors	Conditional variables					Outcome variable
	Self-efficacy	Fear of negative evaluation	Interest	Verbal immediacy	Nonverbal immediacy	Negative silence in classroom
Complete membership point	3.50	4.00	3.00	3.33	3.00	3.75
Intersection point	2.75	2.80	2.00	2.67	2.33	3.00
Complete non-membership point	1.75	1.80	1.33	1.67	1.33	2.00

## Results

### Necessity analysis

Necessity analysis is a necessary process before conducting truth table analysis. If the necessity test value of a certain condition is greater than 0.9, it indicates that the condition is a necessary one for producing the result and needs to be considered in the analysis of the truth table. On the contrary, if it is less than 0.9, it shows that it is not a necessary condition. The necessity test results of conditions in this article are shown in Table 3. From the information in Table 3, none of the conditions is necessary for the phenomenon of negative silence in college classroom.

**Table 3:** Necessity test results of conditional variables.

Conditional variables	Outcome variable
	Negative silence in college classroom
Self-efficacy	0.68
∼Self-efficacy	0.56
Fear of negative evaluation	0.68
∼Fear of negative evaluation	0.58
Interest	0.76
∼Interest	0.49
Verbal immediacy	0.70
∼Verbal immediacy	0.55
Nonverbal immediacy	0.65
∼Nonverbal immediacy	0.60

### Configuration analysis

This article uses FSQCA4.0 software to conduct a configuration analysis of the antecedents that lead to negative silence in college classroom. Different configurations represent combinations of different antecedents that cause the same result. Referring to existing research, this paper sets the thresholds of the number of case, consistency, and PRI (Proportional Reduction in Inconsistency) as 1, 0.75, and 0.6, respectively. The settings of these three parameters are based on references to existing literature, for details, threshold of the number of case is based on the study of Du et al.^73^, threshold of the consistency is based on the study of Schneider and Wagemann^74^, threshold of the PRI is based on the study of Greckhamer^71^. Then, the FSQCA analysis results can be obtained through software, as shown in Table 4.

**Table 4:** Configuration results of high negative silence in college classroom.

Condition	High negative silence in college classroom		
	S1	S2	S3
Self-efficacy	⬤	⬤	⬤
Fear of negative evaluation	⬤	⬤	
Interest			▲
Verbal immediacy	⬤		⬤
Nonverbal immediacy		⬤	⬤
Consistency	0.86	0.87	0.89
Raw coverage	0.43	0.43	0.37
Unique coverage	0.07	0.07	0.04
Overall solution consistency	0.84		
Overall solution coverage	0.54		

Notes: S1 = Set1, S2 = Set2, and S3 = Set3.

This study follows the configuration representation method proposed by Fiss et al.^75^and in Table 4, ⬤ and ▲ indicate the existence of the condition. ⬤ represents a core condition, and ▲ means an edge condition. Blank stands for a condition that can exist or not exist in the configurations with no direct causal relationship to the result. Consistency refers to the degree to which a certain configuration leads to the result. For example, consistency of S1 in Table 4 indicates that when college students have low self-efficacy, are afraid of negative evaluation, and the teacher does not use verbal immediacy in class, the likelihood of negative silence among college students is 86%. Raw coverage indicates the explanatory power of a certain configuration on the result. For instance, raw coverage of S2 in Table 4 means that when college students experience negative silence in class, 43% of the reasons are due to the combined influence of their low self-efficacy, fear of negative evaluation, and the teacher’s nonuse of nonverbal immediacy. Unique coverage implies the unique explanatory power of a configuration on the result. To illustrate, unique coverage of S3 in Table 4 indicates that when college students experience negative silence in the classroom, 4% of the reasons are simply due to the collaborative influence of students’ low self-efficacy, lack of interest, and the teacher’s nonuse of immediacy, which contains both verbal and nonverbal immediacy. Overall solution consistency represents the degree to which all configurations bring about the result. As shown in Table 4, when college students simultaneously meet the conditions in S1, S2, or S3, the likelihood of students being negatively silent in class is 84%. Overall solution coverage refers to the explanatory power of all configurations on the result. As shown in Table 4, when college students exhibit negative silence in classroom, 54% of the reasons are due to the simultaneous occurrence of conditions in S1, S2, or S3.

From the Table 4, three configurations can lead to high negative silence in college classroom. Therefore, Hypothesis 1 and Hypothesis 2 are validated.

(1) S1 configuration demonstrates that regardless of whether college students are interested in class or the teacher uses nonverbal immediacy in classroom, as long as students have poor self-efficacy, fear negative evaluation, and the teacher does not use verbal immediacy, students will show negative silence in classroom. These students are lack of self-efficacy, and they do not believe in their ability to complete the learning of course knowledge. In addition, they worry that their active behaviors in class will receive negative evaluations from other classmates, and they have a mentality of fear towards those. In the meantime, teacher does not use verbal immediate behaviors to attract their attention. As a result, these three reasons work together to make students exhibit negative silence in the classroom.

Through this configuration, it requires both teacher and college students to make efforts to break this negative phenomenon. As the main part of classroom activity, students should work in two aspects that are self-efficacy, fear of negative evaluation. Firstly, self-efficacy represents an individual belief to complete a certain task. Students need gradually improving their self-efficacy, which can help promote them to learn classroom knowledge well. Secondly, eliminating the fear of evaluation requires college students to bravely take a big step forward. They should understand that only by speaking or participating in activities in class can they learn knowledge more effectively. If they are always worried about how others will evaluate their behaviors, the learning effect of knowledge will not be very good. As the guide in classroom, teacher should also use verbal immediate behaviors to break negative silence, such as asking question to students in a timely manner to attract their attention, or breaking existing silence through calling students’ names. Surely, there are also many other verbal immediate behaviors, which the teacher can adopt according to specific situations. This configuration clearly indicates that the insufficient verbal immediate behaviors of teacher is one of the factors that cause negative silence. Therefore, teacher should use effective verbal immediate behaviors in classroom.

(2) S2 configuration implies that as long as college students have low sense of self-efficacy and are afraid of negative evaluation, and teacher’s nonverbal immediacy behavior is not in place, regardless of whether students are interested in the class or teacher uses verbal immediacy, students will show negative silence in class. Compared with S1 configuration, this configuration reflects the importance of teacher’s nonverbal immediacy. To be specific, college students in this configuration are also lack of self-efficacy, and they are afraid that their positive acts in classroom will be negatively evaluated by others. At this time, teacher does not use nonverbal immediate behaviors to attract their attention. Consequently, these students show negative silence during class.

Therefore, it also need to work from both the perspectives of teacher and students. Teacher cannot only give lecture in class. This configuration represents that teacher’s nonverbal immediate behaviors are notable factors related to negative silence in classroom. Therefore, as a crucial component, teacher should use nonverbal immediacies appropriately such as eye contact or gestures because these behaviors can attract students’ attention through visual means. From the perspective of students, self-efficacy needs to be improved. No matter what method is used, students should be confident that they can complete the learning content of class. Although knowledge may seem difficult and complicated to understand, it is all sublimated and accumulated from the most basic content. College students have gone through more than a decade of hard study and mastered the basic knowledge. Therefore, as long as they learn carefully and participate in the class actively, they can definitely master the content of the class well. Even if they haven’t fully understood it in classroom, they can ask the teacher for advices outside of class. It is normal to have fear of negative evaluation, but college students should know that actively answering the teacher’s raised question or being brave enough to participate in classroom discussion is an effective way to exercise themselves. Now it’s the classroom, in the future, it’s the workplace. Developing the will and habit of not being afraid of negative evaluation can better pave the way for their future. At the same time, in this process, students can better grasp knowledge, thereby enhancing the self-efficacy to a certain extent.

(3) S3 configuration shows that the combination of four conditions, namely self-efficacy, interest, verbal immediacy and nonverbal immediacy can lead to negative silence in classroom, regardless of whether students are fear of negative evaluation. Students of this configuration are also have low self-efficacy, and they are not confident with their abilities. Meanwhile, perhaps it is because the knowledge is too difficult to understand, or the knowledge is not attractive to them, they are not interested in class. Additionally, teacher in class does not use verbal or nonverbal immediate behaviors, that is, there are no external factors to attract students’ attention. In such a situation, negative silence occurs.

As with the other two configurations, students’ self-efficacy should be improved undoubtedly. Interest plays a certain role in this configuration, thus, how to enhance students’ interest in the classroom requires thinking and exploration. College students can link the course content with knowledge they have already mastered or are interested in, so as to gradually cultivate their interest. Interest has a good guiding effect, and with it, students will not easily show negative silence. The immediate behaviors of teacher are essential because they can change the absent-minded state of students and attract them to shift their attention to the current classroom. Therefore, teacher must fully use verbal and nonverbal behaviors.

### Robustness analysis

In the process of conducting robustness test, this article first adjusts the consistency threshold from 0.75 to 0.8, and the obtained configuration results are identical to the original results. Then, the PRI consistency threshold is adjusted from 0.6 to 0.65, and then the configuration results are basically the same as the original ones (see Table 5). Therefore, the robustness tests indicate that the configuration results in this paper are robust.

**Table 5:** Robustness test results.

Condition	High negative silence in college classroom (PRI consistency threshold = 0.6)			High negative silence in college classroom (PRI consistency threshold = 0.65)	
	S1	S2	S3	S1’	S2’
Self-efficacy	⬤	⬤	⬤	⬤	⬤
Fear of negative evaluation	⬤	⬤		⬤	
Interest			▲		▲
Verbal immediacy	⬤		⬤		⬤
Nonverbal immediacy		⬤	⬤	⬤	⬤
Consistency	0.86	0.87	0.89	0.87	0.89
Raw coverage	0.43	0.43	0.37	0.43	0.37
Unique coverage	0.07	0.07	0.04	0.10	0.04
Overall solution consistency	0.84			0.87	
Overall solution coverage	0.54			0.47	

Notes: S1 = Set1, S2 = Set2, S3 = Set3, S1’ = Set1’, and S2’ = Set2’.

Since S1, S2, and S3 in Table 5 are identical to those in Table 4, which have been explained earlier, the meanings of consistency, raw coverage, unique coverage, overall solution consistency, and overall solution coverage of S1’ and S2’ in Table 5 are interpreted as follows.

Similar to Table 4, consistency refers to the degree to which a certain configuration leads to the result. For instance, consistency of S1’ in Table 5 indicates that when college students have low self-efficacy, are afraid of negative evaluation, and the teacher does not use nonverbal immediacy in class, the likelihood of students being negatively silent is 87%. Raw coverage indicates the explanatory power of a certain configuration on the result. For instance, raw coverage of S2’ in Table 5 means that when college students experience negative silence in class, 37% of the reasons are due to the combined influence of their low self-efficacy, lack of interest, and the teacher’s nonuse of immediacy, which includes both verbal immediacy and nonverbal immediacy. Unique coverage implies the unique explanatory power of a certain configuration on the result. ‌As an illustration, unique coverage of S1’ in Table 5 indicates that when college students experience negative silence in the classroom, 10% of the reasons are simply due to the collaborative influence of students’ low self-efficacy, fear of negative evaluation, and the teacher’s nonuse of nonverbal immediacy. Overall solution consistency represents the degree to which all configurations bring about the result. As shown in Table 5, when college students simultaneously meet the conditions in S1’ or S2’, the likelihood of students being negatively silent in class is 87%. Overall solution coverage refers to the explanatory power of all configurations on the result. As shown in Table 5, when college students exhibit negative silence in classroom, 47% of the reasons are due to the simultaneous occurrence of conditions in S1’ or S2’.

## Conclusion

This article focuses on 222 college students as research subjects and explores the combined effects of five conditions on negative silence in college classroom with the help of FSQCA method from the perspective of configuration, which could break the limitation of a certain condition’s net effect on the silence in existing literature. The conditions are self-efficacy, fear of negative evaluation, interest, verbal immediacy and nonverbal immediacy. The analysis results indicate that a single condition (self-efficacy, fear of negative evaluation, interest, verbal immediacy, or nonverbal immediacy) is not necessary for negative silence, and there are three configurations that could lead to this phenomenon. The first configuration includes self-efficacy, fear of negative evaluation, and verbal immediacy. The second configuration includes self-efficacy, fear of negative evaluation, and nonverbal immediacy. The third configuration includes self-efficacy, interest, verbal immediacy, and nonverbal immediacy. From this, it can be seen that there are more than one configuration that give rise to this phenomenon. Although these configurations contain different conditions, they can produce the same result, which is the negative silence in college classroom. Through comprehensive comparison of the three configurations, self-efficacy is included in all configurations and appears as the core condition. Fear of negative evaluation, verbal immediacy, and nonverbal immediacy are included as core conditions in two different configurations, and interest appears as an edge condition in one configuration. So, among the five conditions that lead to negative classroom silence among college students, self-efficacy is extremely important. When implementing intervention measures for negative classroom silence, the most focus should be on improving college students’ self-efficacy. The importance of the three conditions of negative evaluation, verbal immediacy, and nonverbal immediacy is second only to self-efficacy. Therefore, it is also necessary to pay much attention to them and take measures in related areas. Although interest is an edge condition, it is indispensable for the third configuration. Therefore, when taking relevant measures, it should also be considered to enhance the interest of college students. In summary, the five conditions appear in different configurations in certain ways, they are related to negative silence of college students in classroom, and attention should be given to all of them so as to break the passive phenomenon.

### Theoretical contributions and practical implications

This article studies the phenomenon of classroom negative silence among college students. Although many studies on it have been achieved rich research results and verified the effects of related factors on this phenomenon^6–11,76^, they are being limited by the relationship between a single factor and result. As a result, FSQCA method basing on configuration thinking is used as a tool to analyze this issue in this paper^54^. Since the occurrence of phenomena should be caused by multiple factors from the perspective of general systems theory, negative silence in college classroom need be discussed from the configuration view, which is actually more in line with the actual situation.

In terms of theoretical contributions: this article adopts FSQCA method to study the negative silence in college classroom and has found that under the five conditions mentioned in this article, three configurations could constitute this issue. Although the three configurations contain different antecedents, the effects achieved by them are equal, which is the manifestation of equivalence of FSQCA method^77,78^. Previous research on this phenomenon has mostly focused on analyzing the net effects of conditions on result, which objectively leads to some fragmented outcomes^79^. FSQCA can be used to integrate these fragments and consider different conditions together. In addition, this study expands the application scope of FSQCA method and applies it to the field of higher education to explore the issue of negative silence in college classroom. The research results obtained can also provide more realistic explanations for this phenomenon and certain theoretical references for in-depth research and subsequent exploration of the phenomenon.

From the data collected during the study, the mean value of negative classroom silence is the highest among all variables at 2.94 (see Table 1), nearly reaching the moderate level (3.00). This indicates that the phenomenon of negative silence in college classroom indeed exists, which aligns with the view of previous literature^80,81^, and also in line with the premise of this article, as the confirmed existence of this phenomenon enables the analysis of its causes. The standard deviation of negative classroom silence (0.72) indicates a good consistency in university students’ manifestation of this phenomenon, with no significant differences among individuals. The standard deviations of self-efficacy, interest, verbal immediacy, and nonverbal immediacy also support this observation. Fear of negative evaluation shows the highest standard deviation (0.90) among all variables, which to some extent reflects variations in individuals’ fear toward negative evaluation^82^. Regarding the maximum values, both interest and nonverbal immediacy reach 4.33, while other four variables have a maximum of 5.00. All variables exhibit a minimum value of 1.00. Thus, the scale designs are relatively reasonable and can distinguish different levels of psychological or behavioral characteristics. From the perspective of necessity, the study reveals that no single condition constitutes a necessary cause of negative classroom silence, meaning individual factors alone cannot trigger such silence. This result aligns with the view of FSQCA method^54^, as well as the research object of this paper, which is exploring condition configurations that lead to negative classroom silence. Regarding the identified configurations, each one includes at least three concurrent conditions, further supporting the view that the phenomenon arises only when multiple factors interact synergistically^13,53^.

In terms of practical implications: negative silence in college classroom is a phenomenon that is not advocated^83^. It negatively affects the learning effectiveness of students, as well as the enthusiasm of teacher in class. Therefore, striving to eliminate it and returning higher education classroom to vibrant state are necessary^84^. In order to achieve this goal, it is noteworthy to understand the reasons, because only by grasping the reasons can corresponding measures be taken to solve it. The results obtained through FSQCA method explain the reasons for this phenomenon, which can provide some bases for corresponding practices. All the three configurations indicate that both students and teacher are the antecedents. Hence, the two levels need to be given priority consideration.

At the student level, it is necessary to adopt reasonable ways to enhance the self-efficacy, overcome the fear of negative evaluation, and cultivate the interest in classroom. Firstly, self-efficacy is essential for college students, as it could determine their classroom learning state, which is also evident from the three configurations. Therefore, effective methods should be used to cultivate it^85^. Everyone has shining points and has experienced some glories and honors, which are materials for enhancing self-efficacy. In addition, college students can choose to do things that are relatively easy in classroom to quickly experience success, which is an effective way to enhance self-efficacy^86^. For instance, sitting in the front row of the classroom during class, listening carefully to the teacher’s lecture, and boldly answering the simple question raised by the teacher that are not difficult. As long as students persist in doing these things seriously, they will definitely be able to understand what the teacher talk. Over time, in situations where students can both understand and answer the teacher’s question, their self-efficacy will be strengthened. Secondly, how to eliminate or reduce fear of negative evaluation still ought to be highly valued. College students should realize that it is normal for them to answer the teacher’s question incorrectly, or their views are not recognized, or even their behaviors are not understood by others. After all, people cannot always be right^87^, and it is also impossible for everyone to be consistent with a certain idea. They just need to be themselves and do their things. Meanwhile, for other students, appropriate guidance should also be provided. For example, it is necessary to let them know that they should respect the views, ideas, and even the behaviors of others^88^. Everyone has the right to evaluate others, but in turn others are likely to evaluate you the same way. Therefore, if students want to receive positive evaluations from others or do not want to get negative evaluations, they should learn how to evaluate others in friendly manners. Moreover, thoughts of people are various^89^. Although your views may not coincide with those of others, it does not mean that you are right and the others are wrong. Thirdly, as is well known, interest is a very considerable factor for college students’ learning^90^. When college students are interested in class, there will be no negative silence to a large extent. The biggest difference between college knowledge and primary and secondary school knowledge is that knowledge in college is more relevant to reality. Therefore, students should strive to combine the knowledge with reality in order to lift their interest in learning. At the same time, from the perspective of teacher, using more attractive PowerPoints or cases related to the knowledge in class can also reinforce college students’ interest to a certain extent. Classroom participation can effectively break the negative silence. According to the research of Karaoglan Yilmaz and Yilmaz^91^, academic motivation is related to classroom participation. As a consequence, providing correct guidance to college students to enhance the academic motivation of them is also of great significance in promoting classroom participation and breaking negative silence.

From the three configurations, it can be seen that both the teacher’s verbal immediacy and nonverbal immediacy play significant roles in the occurrence of negative silence in college classroom. Verbal immediacy can directly and effectively break the negative silence of college students. If teacher ask students question or call on students to answer specific question during the lecture, students’ attention will be focused and they will listen attentively^92^. In addition, teacher should encourage students to participate in discussion sessions in class and then express their discussion results. Discussion requires students to participate, and it can break the passive silence. Besides, this process can deepen students’ understanding of knowledge. Nonverbal immediacy of teacher is equally vital. If teacher suddenly increases the volume during a lecture, it can help students who are not focused break the negative silence. Using corresponding gestures to assist in teaching is also meaningful because when students do not understand the lecture content fully, gestures of the teacher can promote the comprehension. In addition, teacher could move around in the classroom during lecture, rather than always standing on the podium. During the process of walking, students who are in a passive silent state will be motivated to listen attentively when the teacher walks in front of them. Naturally, for some types of students, verbal immediacy may be effective, while for others, nonverbal immediacy is productive. This requires teacher to adopt appropriate immediate behaviors for different students. In short, whether it is verbal immediacy or nonverbal immediacy, teacher should master and use relevant approaches to promote the breaking of negative silence in classroom.

Without doubt, school should also carry out relevant work to break this negative phenomenon in addition to the above two levels. For example, school can provide relevant trainings for teachers to enable them to better understand and effectively adopt immediate behaviors. Active guidance and education should be provided for students as well, which can enable them to have the confidence to overcome difficulties and be more proactive. At the same time, it is equally vital to cultivate students’ interest in the course content through necessary measures, such as related activities and lectures.

Knowledge-sharing means that individuals share and disseminate knowledge to more people through certain channels and methods, thereby enabling knowledge to be infinitely enhanced, developed, enriched, and improved in an atmosphere of communication and sharing^93,94^. Therefore, a good knowledge-sharing atmosphere of learning in the classroom helps to break the negative silence. According to the study of Karaoglan Yilmaz and Yilmaz^95^knowledge-sharing behaviors of college students in the social media based-learning community is significantly related to need for social approval and dispositional hope. So, in the real classroom, these behaviors is also likely to be related to the two aspects. As a result, taking relevant intervention measures from both need for social approval and dispositional hope of students may break negative silence through knowledge-sharing behaviors in classroom. The viewpoint provides valuable references for this article and can be further studied in real-life scenarios. Meanwhile, Internet gaming disorder refers to the long-term desire of young people to play digital games, leading to emotional, mental, behavioral, and living loss of control, and the decline in daily quality of life^96,97^. If college students experience Internet gaming disorder, they may become addicted to online games in the classroom, resulting in negative silence. Previous study has shown that cyberloafing, locus of control, and narcissism are all related to Internet gaming disorder, and this disorder can be reduced by enhancing internal locus of control and decreasing cyberloafing behaviors and narcissism^98^. Therefore, intervention measures can be taken to break negative silence from these three aspects, which is also a valuable direction.

In addition, Karaoglan Yilmaz and Yilmaz studied the issue of digital game addiction among college students and found that selfishness is positively correlated with this addiction^91^, while academic motivation is negatively correlated with it. If students are addicted to digital games, they are likely to play them in class, thus leading to negative silence. So, measures can also be taken from the aspects of selfishness and academic motivation to intervene in digital game addiction, which could useful to break the negative silence. Artificial intelligence has been very developed today, and acquiring artificial intelligence literacy has become increasingly important for individuals in various fields^99^. For this reason, in education field, improving the artificial intelligence literacy of college students as well as teacher is valuable. For instance, it is beneficial to applying related artificial intelligence technologies such as AR and VR in the classroom because they can effectively break negative silence through vivid learning ways^100,101^.

This study employs the FSQCA method to investigate the negative classroom silence among college students in Chinese educational settings. Although previous literature has also indicated that negative silence in college classroom exists across diverse educational contexts^102,103^, whether the findings of this study can be generalized to explain the silence in other different contexts requires further research. For instance, this paper concludes that no single condition alone leads to the negative silence. However, whether a certain factor is necessary for negative silence within other educational settings should be verified with corresponding samples, even if relevant conditions such as self-efficacy and immediacy have been shown to be associated with negative silence within other culture^10,104^. In addition, whether the configurations identified in this paper can be applied to explain the silence in other educational contexts also needs further research. Because the data collected in this paper are based on the Chinese educational background, which is different from other educational settings, then there may be different educational scenarios, teaching processes and learning psychology. As a result, a same phenomenon may be caused by different condition configurations. In summary, the generalizability of this study’s findings to other educational settings necessitates further exploration.

### Limitations and future research directions

This study has the following limitations that should be acknowledged: (1) This article is based on cross-sectional data analysis. Therefore, it cannot determine the causal relationship between variables. For future studies, longitudinal data could be utilized to obtain more in-depth results. (2) The data in this article are all self-reported by participants, so subjective bias is inevitable. For example, participants’ subjective conservative psychology when filling out the questionnaire. As a result, analysis with more objective data is worthy in the future. (3) The research samples of this article comes from Chinese college students, which is a constraint. Considering the differences in cultural backgrounds, whether the research results can be applied to college students in other countries around the world need further exploration. Accordingly, it is meaningful to expand the sample size to get data from other cultural and educational settings, and thus obtain richer results. (4) The measurement tools used in the article are all adapted from other cultural backgrounds, which may have implications for the statistical outcomes. Then, future research will consider using relevant scales from Chinese background to enhance the validity of research outcomes. (5) This study includes a total of 222 participants, and the proportion of female students is larger than that of male students, which may affect the generalizability of the research results in terms of gender. So, using bigger and gender balanced sample to study is also a future direction.

## Electronic supplementary material

Below is the link to the electronic supplementary material.

Supplementary Material 1

Supplementary Material 2

## Data Availability

The data that supported the findings of this study were provided within the supplementary materials.
