# Supplementary material for: Configuration analysis of negative silence in college classroom based on FSQCA method
Source: Sci Rep. 2025 Jul 9;15:24786. doi: 10.1038/s41598-025-09608-5 (PMC12241655; doi:10.1038/s41598-025-09608-5)
Supplement: Supplementary file 2 — Supplementary Material 2 [file 41598_2025_9608_MOESM2_ESM.docx]

| **Variable** | **Item** | **Origin of item** |
| --- | --- | --- |
| Self-efficacy | Item 1. I am not confident that I could deal efficiently with unexpected events. | Steigen, A. M., Finbråten, H. S., & Kleppang, A. L. (2022). Using Rasch Analysis to Assess the Psychometric Properties of a Five-Item Version of the General Self-Efficacy Scale in Adolescents. International Journal of Environmental Research and Public Health, 19(5), 3082. https://doi.org/10.3390/ijerph19053082 |
|  | Item 2. When facing difficulties, I do not think I have good coping capabilities. |  |
|  | Item 3. If someone opposes me, I can’t find the means and ways to get what I want. |  |
|  | Item 4. If I am in trouble, I can’t usually think of a solution. |  |
| Fear of negative  evaluation | Item 5. In class, if I answer the teacher's question or communicate with classmates, I am afraid that they will not approve of me. | Weeks, J. W., Heimberg, R. G., Fresco, D. M., Hart, T. A., Turk, C. L., Schneier, F. R., Liebowitz, M. R., & Strauss, M. E. (2005). Empirical Validation and Psychometric Evaluation of the Brief Fear of Negative Evaluation Scale in Patients With Social Anxiety Disorder. Psychological Assessment, 17(2), 179–190. https://doi.org/10.1037/1040-3590.17.2.179 |
|  | Item 6. In class, if I answer the teacher's question or communicate with classmates, I am afraid that they will find fault with me. |  |
|  | Item 7. In class, if I answer the teacher's question or communicate with classmates, I am usually worried that I will leave a bad impression on them. |  |
|  | Item 8. In class, if I answer the teacher's question or communicate with classmates, I am often worried that I may say the wrong thing. |  |
|  | Item 9. In class, if I answer the teacher's question or communicate with classmates, I am worried about what they may be thinking about me. |  |
| Interest | Item 10. I think the content taught by the teacher in class is not fun. | Kleespies, M. W., Doderer, L., Dierkes, P. W., & Wenzel, V. (2021). Nature Interest Scale - Development and Evaluation of a Measurement Instrument for Individual Interest in Nature. Frontiers in Psychology, 12, 774333.  https://doi.org/10.3389/fpsyg.2021.774333 |
|  | Item 11. I would not want to learn more about the content taught by the teacher in class. |  |
|  | Item 12. The content taught by the teacher in class is not important to me. |  |
| Verbal immediacy | Item 13. The teacher does not use personal examples or talk about experiences he/she has had outside of class. | Gorham, J. (1988). The relationship between verbal teacher immediacy behaviors and student learning. Communication Education, 37(1), 40–53. https://doi.org/10.1080/03634528809378702 |
|  | Item 14. The teacher does not select students by name to answer question in class. |  |
|  | Item 15. There is no collective discussion in class. |  |
| Nonverbal immediacy | Item 16. The teacher does not use any gestures while talking to class. | Gorham, J. (1988). The relationship between verbal teacher immediacy behaviors and student learning. Communication Education, 37(1), 40–53. https://doi.org/10.1080/03634528809378702 |
|  | Item 17. The teacher's vocal expression is monotonous during the class. |  |
|  | Item 18. The teacher does not move around the classroom while teaching. |  |
| Negative silence in  classroom | Item 19. In class, I will not actively respond to the teacher. | Liu, X. (2021). Research on College Students' Negative Silence and Its Formation Mechanism [D]. Nanchang University. |
|  | Item 20. In class, I will not ask the teacher for question. |  |
|  | Item 21. In class, I will not question the views put forward by the teacher. |  |
|  | Item 22. In class, I try not to make eye contact with the teacher. |  |
